# Incidence of Cancer in Shenzhen, Guangdong Province during 2001–2015: A Retrospective Population-Based Study

**DOI:** 10.3390/ijerph14101137

**Published:** 2017-09-27

**Authors:** Zhongyu Xu, Haibin Zhou, Lin Lei, Hongyu Li, Weiye Yu, Zhen Fu, Nanjin Wu, Ji Peng, Ping Yin

**Affiliations:** 1 Department of Epidemiology and Biostatistics and State Key Laboratory of Environment Health, Huazhong University of Science and Technology, Wuhan 430030, China; xuzhongyu1992@sina.com (Z.X.); m201575174@hust.edu.cn (H.L.); FZacadem@outlook.com (Z.F.); m15972949808@163.com (N.W); 2 Shenzhen Center for Chronic Disease Control, Shenzhen 518020, China; zhbsmail@gmail.com (H.Z.); lin.leilana@gmail.com (L.L.); ywy2002@126.com (W.Y.)

**Keywords:** cancer registry, incidence, malignancy, epidemiology, Shenzhen

## Abstract

Cancer is a serious public health issue and the leading cause of death around the world. This article aimed to estimate the cancer incidence and the trend in standardized cancer incidence in Shenzhen, Guangdong province, South China during 2001–2015 by analyzing the cancer data of the population-based cancer registry in Shenzhen. Data were collected from the cancer registry in Shenzhen, which was conducted during 2001–2015. In this registry, the crude incidence rates, age-specific incidence rates, age-standardized incidence rates and cumulative incidence rates were calculated in every five years. Trends for standardized incidence rates of cancers were analyzed by using the joinpoint regression analysis. In total, 33,374.3 thousand person-years (17,593.9 thousand for males and 15,780.4 thousand for females) were monitored over this time period. The number of new cancer cases during 2001–2015 was 59,218 (30,144 and 29,074 for males and females, respectively). The crude incidence during 2001–2005 was 136.44 per 100,000 persons, while the age-standardized rates by Chinese standard population (ASR-China) and by world standard population (ASR-world) were 165.13 and 212.48 per 100,000 persons, respectively. The crude incidence during 2006–2010 was 179.01 per 100,000 persons, while the ASR-China and ASR-world were 168.08 and 214.44 per 100,000 persons, respectively. The crude incidence during 2011–2015 was 196.53 per 100,000 persons, while the ASR-China and ASR-world were 171.44 and 219.99 per 100,000 persons, respectively. During 2001 and 2015, the joinpoint regression analysis showed that the ASR-China of cancer had an overall increase of 0.96% per year and 0.84% per year for males and females respectively, although both of these values (males and females) were non-significant increases. The leading cancer types during 2011–2015 were lung, colorectal, thyroid gland, breast, liver, stomach, cervix, nasopharynx, leukemia and lymphoma. For males, the top five common cancers were lung, liver, colorectal, stomach and thyroid gland. For females, the top five common cancers were breast, thyroid gland, lung, colorectal and cervix. The results of this study showed a heavy cancer burden among the population of Shenzhen, China. Future researches of the etiology and prevention of cancers should be planned in order to reduce the incidence associated with cancers in the future.

## 1. Introduction

Cancer is a serious public health issue with the cancer incidence and mortality increasing year by year. According to a report written by American Cancer Society, cancer is the leading cause of death in the developed countries and the second leading cause of death in the developing countries [1]. In 2012, over 12 million people were diagnosed with cancer and 8 million died due to cancer around the world [2]. As for China, a study reported that the estimates of new cancer cases and cancer deaths in 2010 were three million and two million, respectively [3]. There are some factors that are possibly contributing to the growing incidence and mortality of cancer in developing countries, which include population aging and growth, smoking, physical inactivity, unhealthy diets, environment pollution and occupation exposure [4,5,6]. In particular, the environment pollution, high density population and bad health lifestyles are still serious problems need to be solved in China, which contribute to the obvious increase in the incidence of cancer [7]. Thus, obtaining knowledge about the situation of cancer and controlling the growth in the incidence of cancer are goals that the governments around the world should achieve. Nowadays, cancer reporting and some monitoring projects, such as the GLOBOCAN 2012, have already provided specific estimates of the burden of cancer. Population-based cancer registries are very important in estimating the incidence of cancer in addition to being very effective in the control and prevention of cancer [8]. In Europe, the cancer registries have provided data for the incidence and mortality of cancers for several decades [9], although China has fallen behind in this field. The National Cancer Registry Program was established by the NHFPC in 2008 to collect data, show the cancer incidence and reflect cancer epidemic trends across the country [3].

Shenzhen is a coastal city in Guangdong province in South China, which has experienced a rapid economic development in recent decades. In 1998, Shenzhen Health Bureau established the cancer registry in Shenzhen, which was located in the Shenzhen Center for Chronic Disease Prevention. The implementation of this system of cancer registration and the establishment of the system of cancer reporting was started in June 1998. With more than ten years of experience in cancer registers and the continuous widening of the cancer surveillance, the cancer registration in Shenzhen has played a great role in reporting cancers as well as reflecting the epidemic characteristics and the incidence trend of cancer in Shenzhen. It is also essential to providing the scientific and useful information for the control of cancer in Shenzhen [10].

The aim of study was to present the cancer incidence in Shenzhen during 2001–2015 and show the trend in standardized cancer incidence. In addition, this study could provide insight into the most common types of cancer and show the cancer incidence differences between different age groups and sex. The results of this study could provide information for decision makers in planning measures to prevent and control cancer.

## 2. Materials and Methods

### 2.1. Source of Data

The cancer registry in Shenzhen collected data for patients, who was diagnosed with cancer during 2001–2015. During the period, we collected the data of new cancer cases in Shenzhen, using the International Classification of Diseases (ICD)-10 codes of C00–C97. The cancer registry in Shenzhen has covered all 48 hospitals in Shenzhen that are qualified for cancer diagnosis. Every cancer case from those hospitals will be reported to the cancer registry and thus, every cancer case from registered people and floating people in Shenzhen is included in the data. Information about age and other details are available for the cancer cases. Although the hospitals have collected data on all cancer cases, we only included the cancers among registered people for this study as Shenzhen is an immigrant city and has a large proportion of floating population that cannot be exactly estimated. Thus we only studied the incidence of cancer in the registered population in Shenzhen. During 2001–2015, the total number of new cancer cases in Shenzhen was 59,218 (30,144 and 29,074 for males and females, respectively). The registered population data over the years was obtained from the Shenzhen Statistics Bureau, and the numbers of different age groups (0, 1–4, 5–9, 10–14, ... 85+ years old) and genders or the age distributions of population in different years in Shenzhen were deduced according to the registered population data in each year and the two available age distributions of population in 2000 and 2010 released by Shenzhen Statistics Bureau. The registered population consisted of 33,374.3 thousand person-years (17,593.9 thousand and 15,780.4 thousand for males and females, respectively) during 2001–2015 (Table 1). The incidence of cancers with ICD-10 codes of C00–C97 can be calculated by the data obtained. 

### 2.2. Data Quality Control

The cancer registry in Shenzhen collected the data from lower-level registries and hospitals in every district in Shenzhen, which was recorded by staff and checked for the validity and completeness with exclusion of the duplicate registrations. The validity of the collected data was assessed by the proportion of morphological verification (MV%), the percentage of cancer cases identified with death certification only (DCO%) and the percentage of cancers with undefined or unknown primary sites (O&U%) [11]. Finally, completeness was assessed by the mortality to incidence ratio (M/I) [12].

### 2.3. Statistical Analysis

The crude cancer incidence rate, the age-standardized rate (ASR) by the 1982 Chinese standard population (ASR-China), the worldwide Segi’s population (ASR-world) and cumulative incidence rate (0–74 years old) were calculated every five years. Comparing the different health outcomes between groups or across time periods, where age structures differ, requires techniques that adjust for variations in the age structure of populations [13]. Shenzhen is a new city with a large number of young people swarming into the city in recent decades, so Shenzhen has a younger age structure than most cities in China and around the world. The standard populations are used for calculating age-standardized rates for comparisons. Segi’s “world” population, devised in the late 1950s by cancer epidemiologist Dr Mitsuo Segi, is based on the sum total of male and female populations of the 46 countries in the 1950 publications of the WHO and was adopted by WHO in the mid-1960s for calculating age-standardized rates. It has been widely used in many studies around the world until now [14,15]. As for China, the 1982 Chinese population is widely used as the Chinese standard population for calculating Chinese age-standardized rates as the census work in 1982 was carried out in a comprehensive and systematic manner. Many studies on important diseases in China use this standard population to calculate Chinese age-standardized rates [16]. In addition, the age-specific incidence rates in different age groups were calculated, while the crude cancer incidence rate and the age-standardized rates were analyzed stratified by cancer type, gender and year. Following this, the incidences of the top ten cancers among males and females would be listed respectively. Furthermore, the trends for standardized incidence rates in overall cancer and the top 10 cancers in 2011–2015 for males and females would be analyzed using the joinpoint regression analysis. The 1982 Chinese standard population was used as the standard in the joinpoint analyses. The joinpoint model provides the annual percent changes (APC) of ASR-China of cancers over the years and the detailed information of trends. In this joinpoint regression analysis, the continuous log–linear model with the best fit was selected, while the grid search method was used for the detection of segments that best describe the data. The permutation test was selected as the model selection method to determine the minimum number of ‘‘joinpoints’’. A significance level of 0.05 was used for the permutation test with 4499 of the randomly permuted datasets. The maximum number of “joinpoints” allowed for each analysis was two. The minimum numbers of observations from a joinpoint to either end of the data, and between joinpoints were 3 and 4 respectively, which were the default settings for grid search in Joinpoint4.2.0. The detail theory of the joinpoint model is explained in a previous study [17]. MS Excel 2007 (Microsoft, Redmond, WA, USA), SAS 9.2 (SAS Institute Inc., Cary, NC, USA) and Joinpoint4.2.0 (SEER*Stat, Bethesda, MD, USA) were used for data analysis.

## 3. Results

### 3.1. Data Quality

In the study, a total of 59,218 (30,144 and male and 29,074 female) new cancer cases were collected during 2001–2015. The MV% and DCO% were 75.02% and 1.61%, respectively. The O&U% and the M/I ratio were 2.57% and 0.16, respectively (Table 2).

### 3.2. Incidence Rates

During 2001–2005, the crude incidence of all cancers in the registry was 136.44/100,000 (138.10/100,000 in males and 134.58/100,000 in females). The age-standardized rates by Chinese standard population (ASR-China) and age-standardized rates by world standard population (ASR-world) were 165.13/100,000 and 212.48/100,000, respectively. The cumulative incidence (0–74 years old) rate was 25.39%. During 2006–2010, the crude incidence of all cancers in the registry was 179.01/100,000 (173.23/100,000 in males and 185.56/100,000 in females). The ASR-China and ASR-world were 168.08/100,000 and 214.44/100,000, respectively. The cumulative incidence (0–74 years old) rate was 24.82%. During 2011–2015, the crude incidence of all cancers was 196.53/100,000 (186.47/100,000 in males and 207.56/100,000 in females). The ASR-China and ASR-world were 171.44/100,000 and 219.99/100,000, respectively. The cumulative incidence (0–74 years old) rate was 24.23% (Table 3).

### 3.3. Gender- and Age-Specific Incidence Rates

**Figure 1:**
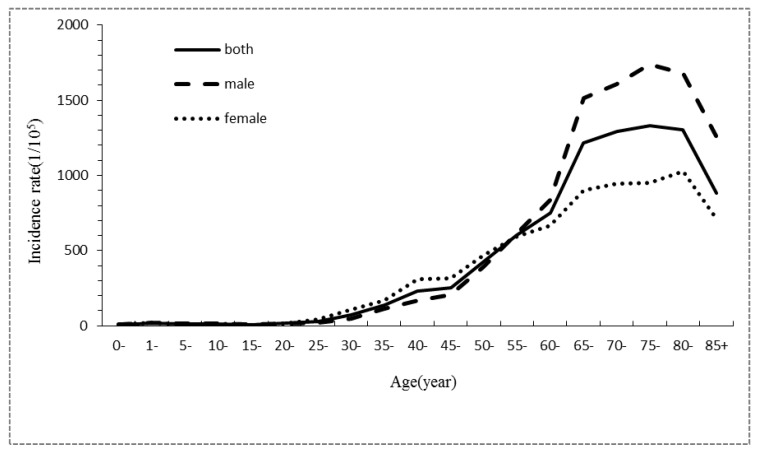
Gender- and age-specific incidence characteristics of all cancer cases registered in Shenzhen during 2001–2005.

**Figure 2:**
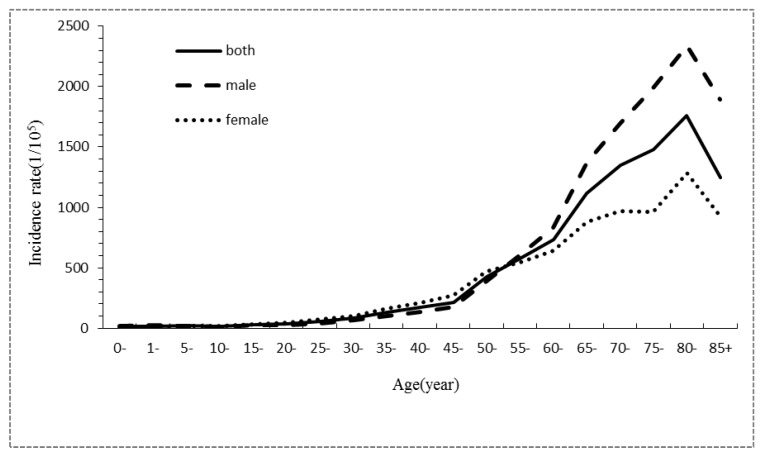
Gender- and age-specific incidence characteristics of all cancer cases registered in Shenzhen during 2006–2010.

**Figure 3:**
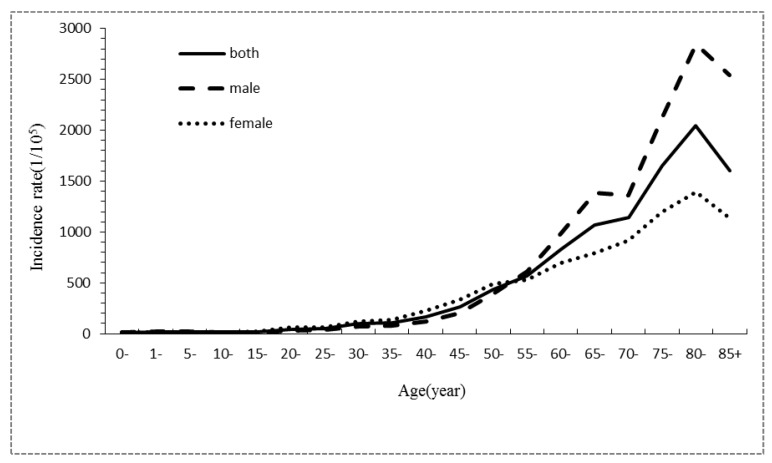
Gender- and age-specific incidence characteristics of all cancer cases registered in Shenzhen during 2011–2015.

During 2001–2005, the age-specific cancer incidence rate was low for individuals <50 years old, before an obvious increase for individuals >50 years old. The highest incidence occurred in individuals of 70–84 years old, before this incidence finally decreased for those over 85 years old. The trends of the age-specific cancer incidence rates during 2006–2010 and 2011–2015 were similar to the trend during 2001–2005, except that the age-specific cancer incidence rate reached the highest value for individuals from 80 to 84 years old. For the three consecutive time periods, the age-specific incidence rates in male were all lower than female for individuals that were 25–54 years old. For individuals over 55 years old, the age-specific incidence rates in males were all much higher than females (Table 4, Figure 1, Figure 2 and Figure 3).

### 3.4. Incidence Rate for Top 10 Common Cancers

During 2001–2005, lung cancer was the most common cancer (CR: 19.92/100,000), followed by colorectal cancer (15.48/100,000), liver cancer (15.37/100,000), breast cancer (13.51/100,000) and stomach cancer (9.89/100,000). The top 10 common cancers accounted for 74.18% of all cancer incidence rates. For males, the top 5 common cancers were lung cancer, liver cancer, colorectal cancer, stomach cancer and nasopharynx cancer. For females, they were breast cancer, colorectal cancer, lung cancer, cervix cancer and thyroid cancer (Table 5).

During 2006–2010, lung cancer was also the most common cancer (25.03/100,000), followed by colorectal cancer (19.80/100,000), breast cancer (18.63/100,000), liver cancer (18.08/100,000) and stomach cancer (10.62/100,000). The top 10 common cancers accounted for 72.83% of all cancer incidence rates. For males, the top 5 common cancers were lung cancer, liver cancer, colorectal cancer, stomach cancer and nasopharynx cancer. For females, they were breast cancer, cervix cancer, lung cancer, colorectal cancer and thyroid cancer (Table 5).

During 2011–2015, lung cancer was also the most common cancer (27.15/100,000), followed by colorectal cancer (22.69/100,000), thyroid cancer (21.01/100,000), breast cancer (21.00/100,000) and liver cancer (16.89/100,000). The top 10 common cancers accounted for 74.35% of all cancer incidence rates. For males, the top 5 common cancers were lung cancer, liver cancer, colorectal cancer, stomach cancer and thyroid gland cancer. For females, they were breast cancer, thyroid gland cancer, lung cancer, colorectal cancer and cervix cancer (Table 5).

For these three time periods, thyroid cancer was the most rapidly rising cancer, which ranked third in 2011–2015. Compared to 2001–2005, the crude incidence of thyroid cancer in 2011–2015 increased by 3.5 times. Liver cancer, nasopharynx cancer and leukemia fell down the ranks in terms of incidence. Furthermore, the rank of prostate cancer rose rapidly, while the rank of esophageal cancer was lower for males. The rank of uterus cancer rose obviously for females. The crude incidences of most cancers rose except for nasopharynx cancer (Table 5).

### 3.5. Analysis of Cancer Incidence Trends during 2001–2015

Results of the ASR-China and joinpoint analyses of top 10 cancers in 2011–2015 in males and females were reported in Table 6 and Table 7.

**Figure 4:**
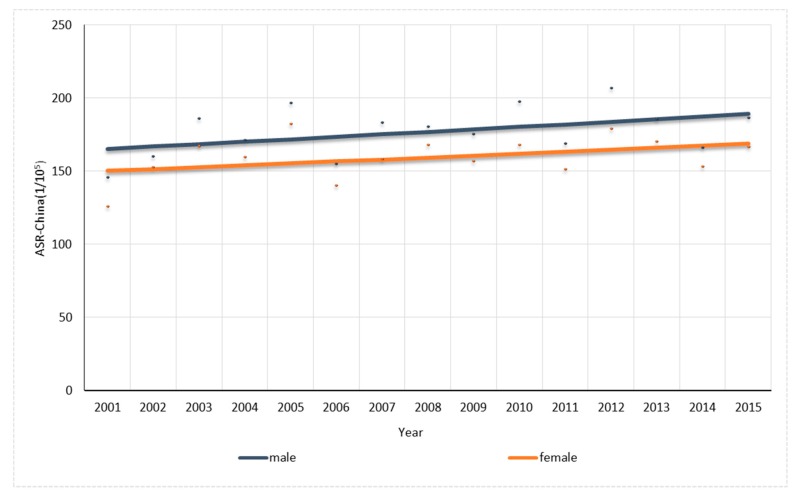
Trend analysis for overall cancer for males and females in Shenzhen.

**Figure 5:**
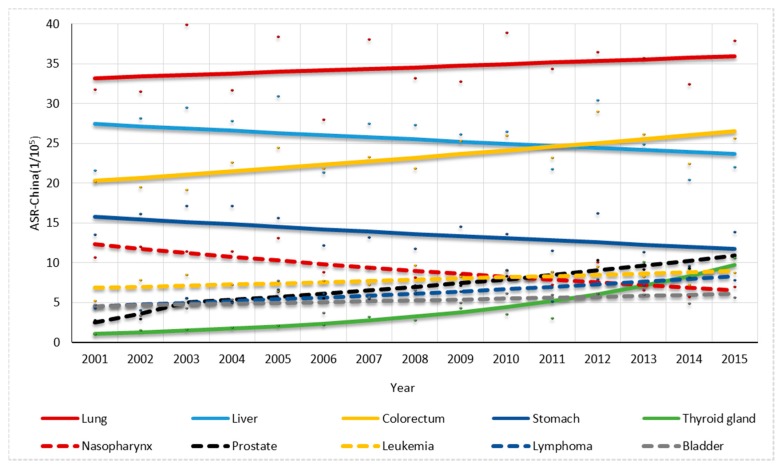
Trend analysis for the top 10 cancer types in 2011–2015 for males in Shenzhen.

For males, one joinpoint was found in the trend in prostate cancer rate while no joinpoint was found in others. The overall ASR-China cancer rate had a non-significant increase of 0.96% per year (Figure 4). The colorectal cancer, thyroid gland cancer, leukemia and lymphoma rates significantly increased by 1.92%, 16.95%, 1.96% and 4.36% per year, respectively, while the stomach cancer and nasopharynx cancer rates significantly decreased by 2.06% and 4.38% per year, respectively. The prostate cancer rate had a significant increase of 41.70% per year by 2003 and also significantly increased thereafter by 6.68% per year (Figure 5).

**Figure 6:**
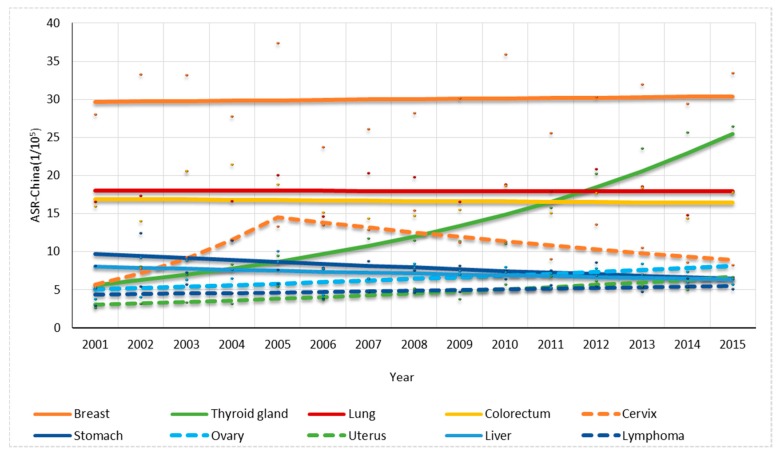
Trend analysis for the top 10 cancer types in 2011–2015 for females in Shenzhen.

For females, one joinpoint was found in the trend in cervix cancer rate while no joinpoint was found in others. The overall cancer ASR-China rate had a non-significant increase of 0.84% per year (Figure 4). The thyroid cancer, ovary cancer and uterus cancer rates increased significantly by 11.38%, 3.42% and 5.78% per year, respectively, while the stomach cancer decreased 2.88% per year significantly. The cervix cancer rate significantly increased up to 2005 by 26.38% per year and significantly decreased thereafter by 4.80% per year (Figure 6).

## 4. Discussion

For the first time, this study presented the incidence of all cancers in recent decades in Shenzhen, the incidence rate distribution in age group, the most common cancers that people suffered from and the trend in standardized cancer incidence by using the data from the cancer registry in Shenzhen and Shenzhen Statistics Bureau. The cancer registry system providing the data was used to calculate the results for the whole city so the study could reflect the actual situation of cancer incidence in Shenzhen. In the study, during these three time periods, the age-specific incidences were all relatively low for individuals <50 years old, increased obviously for individuals >50 years old and reached a peak for individuals around 80–84 years old. The age-specific incidence rates in males were all lower than females for individuals 25–54 years old, while for individuals over 55 years old, the age-specific incidence rates in males were all much higher than females. The results are consistent with those in some studies [3,7,18], which showed that the cancer incidence increased gradually with age and cancer incidence was higher in males than females over 50 years old. The results could be attributed to some risk factors. For examples, males were more likely to have unhealthy and harmful lifestyles, such as smoking and drinking, more than females. The elderly had more possibility to get cancers as they were probably exposed to risk factors for a longer time. For the result that the age-specific incidence rates in females were all higher than males for individuals less than 54 years old, possibly the high incidence rates of breast cancer and cervix cancer for young females could explain this outcome.

The ASR-China of cancer in China in 2011 was 213.66/100,000 and 161.47/100,000 for males and females respectively [19], while the ASR-China of Shenzhen in 2011 was 168.78/100,000 and 151.15/100,000 respectively, which were both lower than the national cancer rates above. The ASR-China of cancer in Guangdong province in 2012 was 229.51/100,000 and 187.82/100,000 respectively [6], while the ASR-China of Shenzhen in 2012 was 206.81/100,000 and 179.07/100,000 respectively. These values are slightly lower than the rates in Guangdong. 

For the comparisons with China [19] in the top ten cancers, lung cancer was the leading cancer among males in Shenzhen, followed by liver cancer, colorectal cancer and stomach cancer. Lung cancer was also the leading cancer among males in China, while stomach cancer was ranked second and esophageal cancer was ranked fourth. It meant that the situation of liver cancer among males was more severe in Shenzhen than in China, while the results were opposite for stomach cancer. For females, breast cancer was the first common cancer in Shenzhen and in China, with the common cancers being similar between in Shenzhen and in China. However, cervix cancer and thyroid cancer were more common in Shenzhen. The differences were likely to be attributed to the local diet habits, different lifestyles and other environmental factors. The results showed that the situation of cancer incidence in Shenzhen was also severe. Shenzhen is known as the first Special Economic Zone in China benefiting from the implementation of the policy of reform and openness since 1979. Within a few decades, there has been rapid developments in economy as well as people’s quality of life and spiritual civilization, which has marked Shenzhen as a dynamic and modernized metropolis in China [20]. However, there are also some bad effects accompanying this rapid development of the economy, such as the high-density population and bad health lifestyles. With the fast pace of life and the increasing competition for jobs, people are more likely to have bad lifestyle habits. Unhealthy diets, such as eating salty foods and low intake of vegetable and fruit, are the clear or probable risk factors for cancer. The AICR/WCRF (American Institute for Cancer Research/ World Cancer Research Fund) reports a probable decreased risk between the following: non-starchy vegetables with stomach cancer; and fruits with lung and stomach cancer. A probable increased risk exists between the following: Cantonese-style salted fish with nasopharyngeal cancer; and salty foods with stomach cancer [21]. Aflatoxin found in maize and peanuts is a clear risk factor for liver cancer [22]. A research in Shenzhen shows that the positive ratio of aflatoxin in corn flour is 46.7% and the over standard rate of aflatoxin B1 is 5.66% as excessive samples are all self-pressed peanut oil [23]. Besides, some diseases are also the important risk factors for cancer. For example, HBV (Hepatitis B virus) and HCV (Hepatitis C virus) infections play the most important roles in liver carcinogenesis [24]. Researches show the incidence of Hepatitis B in Shenzhen in 2013–2015 is 79.4/100,000 with HBsAg (Hepatitis B virus surface antigen) positive rate of 9.48% and the incidence of hepatitis C shows an upward trend in Shenzhen [25,26]. As for obesity, lack of physical exercise, drinking and smoking, a survey conducted in Nanshan district in Shenzhen shows the prevalence rates of overweight and obesity are 30.3% and 7.4% respectively; and the prevalence rate of drinking is 62.7%. Besides, the smoking rate of male adult is 21.6% and only 50.2% of residents exercise in spare time [27]. It is found that lacking physical activity and obesity are important risk factors for cancer [28,29]. In terms of alcohol and smoking, a meta-analysis shows that tobacco usage, particularly smoking, is a heavily weighted risk factor for multiple types of cancer, such as lung and pharyngeal cancers [30]. Another meta-analysis has shown that a causal association has been established between alcohol consumption and pharynx, liver, colorectal and breast cancers [31]. Moreover, outdoor air pollution and airborne particulate matter are classified as Group 1 lung carcinogens in humans by the International Agency for Research on Cancer and a research shows air concentrations of nitrogen oxides and inhalable particle matters increase fast in Shenzhen in 2002–2004 [32,33]. Besides, having multiple sexual partners and early onset of sexual intercourse are known as strong risk factors for cervix cancer as Shenzhen is a modern city with a comparatively open attitude to sex [34]. 

For the trends in cancer incidence in Shenzhen, the result in this study showed a slight increase in the standardized rate for overall incidence of cancer, while the standardized rates for nasopharynx cancer among males as well as liver and stomach cancer among males and females decreased. Possibly, the implement of health education and health promotion could lead to the results. Since 1996, Shenzhen government and Shenzhen Health Bureau have promulgated a series of regulations and policies to guarantee health education and health promotion. In 2001, Shenzhen government, the Shenzhen Health Bureau and Health institution founded the “health promoting school” and created the “community health education model” in health education and health promotion work. Healthy lifestyles are advocated by distributing health brochures, holding subject lectures and media propaganda [35]. The standardized rates for colorectal cancer among males increased, with the westernized diet possibly being able to explain the result. Some studies showed that pickled and baked food, and increased intake of meat can lead to colorectal cancer [36,37]. Interestingly, the standardized rates for thyroid cancer among males and females increased rapidly. In fact, increasing incidence rates of thyroid cancer have been seen in many studies. For example, a study showed that the age-standardized incidence rate of thyroid cancer increased by 3.1% and 3.8% per year among males and females in Shanghai during the period of 1973–2009 [38]. This could be attributed to a combination of the implementation of more sensitive diagnostic techniques, such as CT (Computed Tomography), MRI (Magnetic Resonance Imaging) and PET (Positron Emission Computed Tomography) scan, and the increasing prevalence of ionizing radiation exposure and occupational exposure to organic solvents in the populations [39]. The standardized rates for prostate cancer also increased rapidly, which was consistent with a study in 2015 reporting that prostate cancer incidence increased steadily over the last decade in East Asia. This change was attributed to the westernized diet and lifestyle and the use of screening as the PSA testing has been conducted since the 1990s in China including Shenzhen [40]. As the rate of cervix cancer increased during 2001–2005 and then decreased in the following years, some studies also showed a declining trend in cervix cancer [41]. This could be partly explained by the public health interventions as Shenzhen has devoted itself to a focus on cervix cancer prevention since 2005. In 2005, Shenzhen became the demonstration base of cervical cancer prevention and control in China. To raise people's awareness about cervical cancer prevention and control, health education of cervical cancer has been conducted in many communities and the cervical cancer prevention and control network has been established and improved since then. In addition, cervical cancer screening has also been planned and conducted to prevent health community residents from cervical cancer [42]. It shows the necessity of the implementation of public health programs to increase the coverage of cervical cancer screening and health interventions to deal with cervix cancer [43].

The study is also confronted with some limitations. First, there are few similar studies in Shenzhen with which we can compare our data to examine the accuracy of the study. Second, the data in the registry was filled in by staff and errors in filling in the register are inevitable. Moreover, for lack of the age structure in every year, the age structures in some certain years were calculated by the existing age structures, which could lead to a calculation error in the result.

## 5. Conclusions

Overall, the results from the study showed that there is a heavy cancer burden in Shenzhen and cancer is more likely to occur in elderly people, and is more common among males. Lung, colorectal, thyroid gland, breast, liver and stomach tumors are the main cancers affecting Shenzhen residents. For this period, the ASR-China cancer rate in Shenzhen had a small and non-significant increase. Based on the results, the government should take measures in health education, protecting healthy living environments and prompt screening of chronic diseases. Future population-based epidemiological studies and research into the etiology of cancers should be conducted to further study the trends associated with cancers and potential risk factors for cancers.

## Figures and Tables

**Table 1:** Registered population coverage in Shenzhen city during 2001–2015 (×10^3^ persons).

Year	Total	Male	Female
2001	1284.8	673.4	611.4
2002	1357.5	715.3	642.2
2003	1452.0	766.6	685.4
2004	1580.3	836.5	743.8
2005	1735.4	921.4	814.0
2006	1893.8	1007.2	886.6
2007	2046.1	1088.7	957.4
2008	2202.3	1171.0	1031.3
2009	2347.6	1248.0	1099.6
2010	2462.4	1308.0	1154.5
2011	2594.7	1374.1	1220.6
2012	2777.6	1463.8	1313.8
2013	2990.5	1565.4	1425.1
2014	3213.4	1670.5	1543.0
2015	3436.0	1784.1	1651.9
total	33,374.3	17,593.9	15,780.4

**Table 2:** Quality evaluation of cancers of every site in Shenzhen cancer registration during 2001–2015.

Cancer	ICD-10	MV (%)	DCO (%)	M/I
Oral & pharynx	C00–C10, C12–C14	89.03	0.68	0.12
Nasopharynx	C11	81.56	1.47	0.12
Esophagus	C15	78.99	1.45	0.19
Stomach	C16	81.70	1.87	0.20
Colorectal	C18–C21	86.82	1.03	0.15
Liver	C22	31.74	2.69	0.30
Gallbladder & extrahepatic bile duct	C23–C24	46.80	1.88	0.29
Pancreas	C25	34.62	6.00	0.36
Lung	C33–C34	55.28	2.74	0.28
Bone	C40–C41	63.67	1.13	0.21
Breast	C50	93.00	0.50	0.05
Cervix	C53	93.09	0.68	0.06
Uterus	C54–C55	93.98	0.77	0.06
Ovary	C56	83.94	1.27	0.14
Prostate	C61	73.13	1.31	0.15
Kidney & unspecified urinary organs	C64–C66, C68	71.88	1.39	0.11
Bladder	C67	82.24	0.54	0.09
Brain & CNS (Central nervous system)	C70–C72	59.97	2.11	0.17
Thyroid gland	C73	96.00	0.09	0.01
Lymphoma	C81–C85, C88, C90, C96	91.74	1.79	0.19
Leukemia	C91–C95	95.36	2.71	0.20
Total	ALL	75.02	1.61	0.16

MV% = percentage of morphological verification; DCO% = percentage of cancer cases identified with death certification only; and M/I = mortality to incidence ratio.

**Table 3:** The incidence rate of cancers (ICD-10:C00-C97) registered in Shenzhen during 2001–2015.

Year	Sex	No. of Cases	Incidence Rate (1/10^5^)	ASR-China ^†^ (1/10^5^)	ASR-World ^§^ (1/10^5^)	Cumulative Rate 0–74 Years Old (%)
2001–2005	both	10,110	136.44	165.13	212.48	25.39
	male	5404	138.10	173.65	227.60	27.93
	female	4706	134.58	159.16	200.77	22.84
2006–2010	both	19,605	179.01	168.08	214.44	24.82
	male	10,087	173.23	179.31	234.42	27.56
	female	9518	185.56	159.03	198.21	22.17
2011–2015	both	29,503	196.53	171.44	219.99	24.23
	male	14,653	186.47	182.70	241.15	26.70
	female	14,850	207.56	164.10	205.50	22.13

^†^ = age-standardized incidence rate (China population, 1982); and ^§^ = age-standardized incidence rate (Segi’s population).

**Table 4:** Gender- and age-specific incidence characteristics of all cancer cases registered in Shenzhen during 2001–2015.

Age Groups (Years)	2001–2005 Incidence Rate (1/10^5^)			2006–2010 Incidence Rate (1/10^5^)			2011–2015 Incidence Rate (1/10^5^)		
	Both	Male	Female	Both	Male	Female	Both	Male	Female
0–	8.60	9.98	6.82	14.83	15.76	13.74	12.51	11.05	14.13
1–	18.91	18.86	18.68	18.01	23.33	11.70	20.15	22.96	17.09
5–	13.58	14.81	11.59	18.14	19.84	15.99	15.74	18.18	12.94
10–	12.65	11.98	13.40	13.24	11.82	15.09	13.99	15.91	11.61
15–	6.95	9.61	5.69	25.60	22.76	29.23	20.44	17.04	25.36
20–	14.70	16.37	13.99	31.95	22.88	43.20	42.19	29.04	62.04
25–	28.59	19.22	40.88	53.38	39.03	69.98	51.19	37.63	66.34
30–	74.52	49.05	106.40	82.74	62.97	102.88	96.13	74.56	116.32
35–	137.20	111.60	165.77	132.79	103.33	163.06	107.05	79.81	134.25
40–	229.61	166.60	308.00	173.09	137.50	212.68	168.68	118.75	224.66
45–	253.30	206.94	313.92	216.03	172.14	269.72	261.95	200.55	332.35
50–	427.03	391.78	468.24	424.40	389.61	466.93	436.49	389.36	490.78
55–	605.54	615.55	596.29	577.12	606.74	547.31	570.68	616.27	526.85
60–	752.68	833.20	664.08	734.22	831.75	637.89	831.47	978.78	695.61
65–	1214.37	1516.53	898.73	1116.96	1374.09	877.65	1070.32	1386.22	795.42
70–	1290.88	1604.85	944.17	1346.36	1695.04	969.92	1140.06	1357.17	915.46
75–	1333.62	1738.17	952.10	1482.23	1991.92	965.41	1654.14	2119.49	1193.65
80–	1306.35	1680.00	1026.98	1757.22	2335.09	1284.95	2044.78	2850.08	1389.61
85+	880.02	1261.90	713.76	1244.98	1892.51	930.56	1599.95	2539.70	1136.80

**Table 5:** Crude incidences of the top 10 cancers in Shenzhen during 2001–2015.

Sex	Rank	2001–2005			2006–2010			2011–2015			
		Site	Incidence (1/10^5^)	Proportion (%)	Site	Incidence (1/10^5^)	Proportion (%)	Site	Incidence (1/10^5^)	Proportion (%)	Percent Change in Incidence vs. 2001–2005
both	1	Lung	19.92	14.60	Lung	25.03	13.98	Lung	27.15	13.82	36.30%
	2	Colorectal	15.48	11.35	Colorectal	19.80	11.06	Colorectal	22.69	11.55	46.58%
	3	Liver	15.37	11.27	Breast	18.63	10.41	Thyroid gland	21.01	10.69	348.93%
	4	Breast	13.51	9.90	Liver	18.08	10.10	Breast	21.00	10.68	55.44%
	5	Stomach	9.89	7.25	Stomach	10.62	5.93	Liver	16.89	8.60	9.89%
	6	Nasopharynx	7.84	5.75	Thyroid gland	8.94	4.99	Stomach	10.52	5.36	6.37%
	7	Leukemia	5.72	4.19	Cervix	8.91	4.98	Cervix	6.94	3.53	51.53%
	8	Thyroid gland	4.68	3.43	Nasopharynx	7.70	4.30	Nasopharynx	6.84	3.48	–12.76%
	9	Cervix	4.58	3.35	Leukemia	7.04	3.93	Leukemia	6.52	3.32	13.99%
	10	Esophagus	4.21	3.09	Lymphoma	5.64	3.15	Lymphoma	6.52	3.32	59.41%
male	1	Lung	25.25	18.28	Lung	30.72	17.74	Lung	33.28	17.85	31.80%
	2	Liver	23.25	16.84	Liver	27.27	15.74	Liver	25.71	13.79	10.58%
	3	Colorectal	16.46	11.92	Colorectal	22.36	12.91	Colorectal	25.40	13.62	54.31%
	4	Stomach	11.93	8.64	Stomach	12.24	7.07	Stomach	12.27	6.58	2.85%
	5	Nasopharynx	11.32	8.20	Nasopharynx	10.66	6.16	Thyroid gland	11.44	6.14	576.92%
	6	Leukemia	6.11	4.42	Leukemia	7.88	4.55	Nasopharynx	9.39	5.04	–17.05%
	7	Esophagus	5.72	4.15	Lymphoma	6.25	3.61	Prostate	8.55	4.59	161.47%
	8	Lymphoma	4.17	3.02	Prostate	5.94	3.43	Leukemia	7.94	4.26	29.95%
	9	Bladder	3.99	2.89	Esophagus	5.86	3.38	Lymphoma	7.32	3.92	75.54%
	10	Brain& CNS	3.58	2.59	Bladder	5.15	2.97	Bladder	5.70	3.06	42.86%
female	1	Breast	28.57	21.23	Breast	39.42	21.24	Breast	43.72	21.06	53.03%
	2	Colorectal	14.38	10.69	Cervix	19.03	10.25	Thyroid gland	31.52	15.19	292.04%
	3	Lung	13.96	10.37	Lung	18.56	10.00	Lung	20.42	9.84	46.28%
	4	Cervix	9.69	7.20	Colorectal	16.88	9.10	Colorectal	19.72	9.50	37.13%
	5	Thyroid gland	8.04	5.97	Thyroid gland	14.33	7.72	Cervix	14.56	7.02	50.03%
	6	Stomach	7.61	5.65	Stomach	8.77	4.73	Stomach	8.61	4.15	13.14%
	7	Liver	6.55	4.87	Ovary	7.93	4.28	Ovary	8.34	4.02	62.89%
	8	Leukemia	5.29	3.93	Liver	7.64	4.12	Uterus	7.60	3.66	165.73%
	9	Ovary	5.12	3.80	Leukemia	6.08	3.28	Liver	7.21	3.47	10.08%
	10	Lymphoma	4.00	2.97	Uterus	5.24	2.83	Lymphoma	5.65	2.72	41.25%

**Table 6:** Trend analysis for the top 10 cancer types in 2011–2015 in Shenzhen during 2001–2015 (males).

ASR-China (1/10^5^) ^†^											
Year	All cancers	Lung	Liver	Colorectal	Stomach	Thyroid Gland	Nasopharynx	Prostate	Leukemia	Lymphoma	Bladder
2001	145.79	31.75	21.57	20.15	13.50	0.95	10.64	2.76	5.23	4.24	4.69
2002	159.91	31.54	28.15	19.51	16.09	1.53	12.02	2.94	7.82	3.99	3.36
2003	185.98	39.90	29.45	19.15	17.17	1.59	11.39	5.52	8.46	5.55	4.25
2004	171.20	31.68	27.79	22.60	17.17	1.85	11.42	5.12	7.18	4.95	5.36
2005	196.65	38.37	30.88	24.42	15.62	2.12	13.10	6.33	6.45	6.61	7.70
2006	154.95	27.99	21.36	21.80	12.20	2.21	8.77	5.57	7.71	6.10	3.70
2007	183.18	38.08	27.45	23.29	13.21	3.19	9.28	6.48	7.17	5.44	5.63
2008	180.45	33.18	27.31	21.84	11.77	2.77	8.12	7.12	9.66	6.08	6.52
2009	175.32	32.78	26.11	25.25	14.55	4.30	8.45	7.09	8.24	6.41	4.93
2010	197.65	38.91	26.45	25.92	13.58	3.50	8.97	8.29	8.96	9.10	6.13
2011	168.78	34.32	21.75	23.17	11.53	2.97	7.22	8.85	8.85	5.13	5.98
2012	206.81	36.44	30.43	29.02	16.22	6.06	10.04	10.34	9.46	7.92	6.11
2013	185.53	35.73	24.89	26.13	11.36	10.05	6.58	9.15	8.26	7.16	5.92
2014	166.05	32.47	20.41	22.44	9.57	9.20	5.66	9.28	7.18	8.15	4.88
2015	186.72	37.91	21.98	25.59	13.85	10.60	7.00	10.97	8.73	7.84	5.58
APC (%) ^‡^	0.96	0.57	–1.05	1.92 *	–2.06 *	16.95 *	–4.38 *	41.70 * (2001–2003)	1.96 *	4.36 *	2.08
								6.68 * (2003–2015)			

^†^ = age-standardized incidence rate (China population, 1982); ^‡^ = annual percent change of ASR-China; and * The APC is significantly different from zero at alpha = 0.05.

**Table 7:** Trend analysis for the top 10 cancer types in 2011–2015 in Shenzhen during 2001–2015 (females).

ASR-China (1/10^5^) ^†^											
Year	All cancers	Breast	Thyroid Gland	Lung	Colorectal	Cervix	Stomach	Ovary	Uterus	Liver	Lymphoma
2001	125.87	27.98	5.16	16.57	15.96	5.36	8.10	3.71	2.64	5.14	2.87
2002	152.52	33.22	8.06	17.35	13.98	7.74	12.42	4.04	3.15	9.19	5.38
2003	166.99	33.17	7.01	20.52	20.60	9.25	7.28	6.29	3.34	8.79	5.65
2004	159.73	27.75	8.34	16.65	21.40	11.43	11.46	6.46	3.16	7.59	4.54
2005	182.29	37.37	9.46	20.04	18.77	13.29	7.64	8.61	5.38	10.07	5.73
2006	140.33	23.70	7.70	14.62	15.10	13.44	7.82	4.30	3.67	7.72	3.84
2007	158.17	26.05	11.69	20.30	14.37	12.87	8.74	6.43	4.59	6.38	4.74
2008	167.89	28.15	11.45	19.78	14.71	15.41	7.54	8.39	5.19	7.84	4.65
2009	157.05	30.10	11.37	16.56	15.44	11.18	8.11	7.01	3.73	7.45	4.69
2010	168.08	35.87	11.04	18.82	18.59	11.29	7.49	7.99	5.69	7.25	6.36
2011	151.15	25.51	15.71	17.90	15.07	8.96	7.53	7.04	6.70	5.22	5.58
2012	179.07	30.30	20.23	20.80	17.71	13.54	8.58	7.75	6.08	6.80	5.42
2013	170.32	31.91	23.51	18.53	18.41	10.46	5.36	8.40	5.54	6.98	4.68
2014	153.41	29.36	25.65	14.75	14.33	8.59	6.45	7.35	5.01	5.90	5.36
2015	166.40	33.42	26.40	17.82	17.72	8.21	6.67	5.64	6.65	6.38	5.07
APC (%) ^‡^	0.84	0.17	11.38 *	–0.04	–0.22	26.38 * (2001–2005)	–2.88 *	3.42 *	5.78 *	–1.66	1.67
						–4.80 * (2005–2015)					

^†^ = age-standardized incidence rate (China population, 1982); ^‡^ = annual percent change of ASR-China; and * The APC is significantly different from zero at alpha = 0.05.
